# Gefitinib facilitates PINK1/Parkin-mediated mitophagy by enhancing mitochondrial recruitment of OPTN

**DOI:** 10.1016/j.fmre.2021.12.017

**Published:** 2022-03-03

**Authors:** Ningning Li, Shan Sun, Guoqiang Ma, Hongyu Hou, Qilian Ma, Li Zhang, Zengli Zhang, Hongfeng Wang, Zheng Ying

**Affiliations:** a Jiangsu Key Laboratory of Neuropsychiatric Diseases and College of Pharmaceutical Sciences, Soochow University, Suzhou, Jiangsu 215123, China; b Department of Respiratory and Critical Care Medicine, The Second Affiliated Hospital of Soochow University, Suzhou, Jiangsu 215004, China; c Key Laboratory of Nuclear Medicine, Ministry of Health, Jiangsu Key Laboratory of Molecular Nuclear Medicine, Jiangsu Institute of Nuclear Medicine, Wuxi, Jiangsu 214063, China

**Keywords:** Gefitinib, Autophagy, Mitophagy, Parkin, OPTN

## Abstract

Gefitinib, a well-known epidermal growth factor receptor (EGFR) tyrosine kinase inhibitor for the targeted therapy of lung cancer, induces autophagy in association with drug resistance. However, it remains unclear whether gefitinib treatment can affect the selective form of autophagy (i.e., mitophagy) and be beneficial for the treatment of human diseases with decreased autophagy, such as neurodegenerative diseases. Here, we show that gefitinib treatment promotes PINK1/Parkin-mediated mitophagy in both nonneuronal and neuronal cells, and this effect is independent of EGFR. Moreover, we found that gefitinib treatment increases the recruitment of the autophagy receptor optineurin (OPTN) to damaged mitochondria, which is a downstream signaling event in PINK1/Parkin-mediated mitophagy. In addition, gefitinib treatment significantly alleviated neuronal damage in TBK1-deficient neurons, resulting in impeded mitophagy. In conclusion, our study suggests that gefitinib promotes PINK1/Parkin-mediated mitophagy via OPTN and may be beneficial for the treatment of neurodegenerative diseases that are associated with defective mitophagy.

## Graphical abstract

**Figure:**
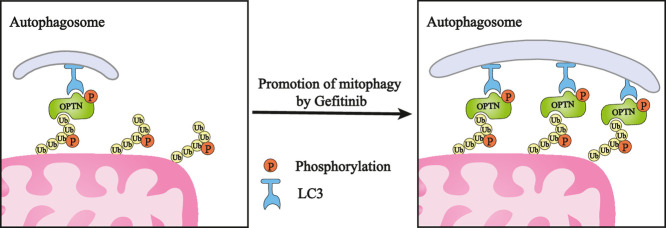

## 1 Introduction

PINK1/Parkin-mediated mitophagy is a well-known form of selective autophagy that targets damaged or unwanted mitochondria for lysosomal degradation and is associated with multiple human diseases, especially neurodegenerative diseases [1]. In an intact PINK1/Parkin-mediated mitophagy signaling pathway, the ubiquitin kinase PINK1 phosphorylates ubiquitin to initiate mitophagy [2]. The interaction between phosphorylated ubiquitin and the Parkin RING1 domain leads to a conformational change in Parkin, which enables phosphorylation of the released Parkin UBL domain and further activation of Parkin [3]. Fully activated Parkin promotes the ubiquitination of outer mitochondrial membrane (OMM) proteins [2,4]. The downstream signaling of mitophagy includes the recruitment of autophagy receptors and autophagosomes. Previous studies have shown that optineurin (OPTN) is the key mitophagy receptor that can bind to the polyubiquitin chain on the OMM and ATG8 family proteins, including LC3, on autophagosomes, thereby recruiting autophagosomes to damaged mitochondria [5,6]. During this step, phosphorylation of OPTN is essential for LC3-positive autophagosome formation around damaged mitochondria, and TBK1 has been identified as an OPTN kinase that phosphorylates OPTN on multiple sites in association with autophagy [7], [8], [9]. The abovementioned mitophagy signaling is remarkably robust and can ensure the complete clearance of damaged mitochondria within 2 days in cultured cells [10].

Notably, decreased mitophagy is widely and tightly associated with neurodegenerative diseases, including Parkinson's disease (PD) and amyotrophic lateral sclerosis (ALS), since loss of function mutations in Parkin (gene product of *PARK2*) and PINK1 (gene product of *PARK6*) cause PD, and loss of function mutations in OPTN (gene product of *ALS2*) and TBK1 (gene product of *ALS-FTD4*) cause ALS [1,11]. However, pharmacological approaches that can activate mitophagy are limited and usually cannot be considered clinically useful candidates simply because these conventional approaches to induce mitophagy are mitochondrial toxins [12]. For example, protonophore and mitochondrial respiratory chain inhibitors, which dissipate mitochondrial membrane potential and stabilize the mitophagy initiator PINK1, are widely used in the study of mitophagy in cellular models [12]. Therefore, there is a need to identify novel candidate drugs with pharmacological targets that function downstream of PINK1/Parkin in the mitophagy signaling pathway. It will be particularly important if the candidate drug can affect the recognition of mitochondria by autophagy without harming mitochondria.

In this study, we show that gefitinib, an inhibitor of epidermal growth factor receptor (EGFR) that is widely used for clinical therapy of non-small-cell lung cancer (NSCLC), strikingly enhances PINK1/Parkin-mediated mitophagy. Interestingly and unexpectedly, gefitinib-induced mitophagy does not depend on EGFR but is associated with enhancement of mitochondrial recruitment of OPTN, the downstream player in the PINK1/Parkin-mediated mitophagy signaling pathway. Importantly, we found that treatment with 50 nM gefitinib (low dose) significantly reduced neuronal damage in a TBK1-deficient cellular model of ALS, indicating that pharmacological modulation of OPTN in mitophagy might be a new strategy for the treatment of ALS and possibly other neurodegenerative disorders.

## 2 Materials and methods

### 2.1 Plasmid constructs and siRNAs

The critical plasmids for this study, including the empty vectors expressing the Tag, mCherry-Parkin, BFP-Mito, mt-mKeima, mCherry-LC3 (LC3B), mCherry-EGFP-LC3 (LC3B), GFP-OPTN, GFP-OPTN E478G, GFP-Parkin, HA-OPTN, FLAG-Parkin, FLAG-TBK1, FLAG-TBK1 E696K, FLAG-TBK1 ∆690-713 and GFP-Mito were described previously [13], [14], [15], [16], [17], [18]. All the plasmids used in this study were verified by sequencing (Synbio Technologies). siRNAs targeting human *EGFR* (5’-CGCAAAGUGUGUAACGGAAUA-3’), human *PINK1* (5’-GGACGCUGUUCCUCGUUAU-3’) and human *TBK1* (5’-CAGAACCCGCACCACUGUUAUA-3’) were synthesized by GenePharma. siRNA targeting human *OPTN* was described previously [13]. siRNAs targeting mouse *Tbk1* and *Optn* were synthesized by GenePharma.

### 2.2 Cell culture, transfection and chemicals

Human embryonic kidney 293 (HEK293) cells, Mouse Embryonic Fibroblast (MEF) cells, and A549 cells were cultured in Dulbecco's modified Eagle's medium (DMEM; Gibco) or RPMI-1640 medium (Gibco) supplemented with 10% fetal bovine serum (FBS; Gibco) with 100 U/ml penicillin and 100 μg/ml streptomycin (Gibco) and kept at 37°C in 5% CO_2_ incubator. The plasmids were transfected into cells with HieffTrans^TM^ Liposomal Transfection Reagent (Yeasen) in Opti-MEM (OMEM; Gibco) without serum and siRNA were transfected using RNAiMAX reagent (Invitrogen) with serum. Primary cortical neurons were obtained from E18-20 mouse embryos and cultured in neurobasal medium (Gibco) with sodium pyruvate (Life Technologies), B27 Supplement (Gibco), Hepes (Sigma) and glutamax (Life Technologies) for 7-10 days. mt-mKeima was expressed in cortical neurons through lentivirus infection. The following drugs were used in the present study: gefitinib (MedChemExpress), Bafilomycin A1 (Selleck), antimycin A (Sigma) and oligomycin A (Selleck). For the drug treatments, DMSO (BBI Life Science Corporation) was used as the control.

### 2.3 qPCR

Total RNA in HEK293 cells was isolated with RNA isolater total RNA extraction reagent (Vazyme). cDNA was obtained using HiScrip III RT SuperMix for qPCR (Vazyme) and then ChamQ universal SYBR qPCR Master Mix (Vazyme) was used to detect the mRNA level of target gene. The following primers were used: *EGFR*-human 5’-ATGCTCTACAACCCCACCAC-3’ and 5’-GCCCTTCGCACTTCTTACAC-3’, *GAPDH*-human: 5′-AAATCCCATCACCATCTTCCAG-3’ and 5’-AGGGGCCATCCACAGTCTTCT-3’.

### 2.4 Immunoblot

The cells were collected after drug treatments for indicated times and lysed in cell lysis buffer (50 mM Tris–HCl (pH 7.6) with protease inhibitor cocktail (Roche), 0.5% sodium deoxycholate, 1% NP-40 and 150 mM NaCl). Proteins were separated by 10% or 12% polyacrylamide gel electrophoresis (SDS–PAGE) and transferred onto polyvinylidene difluoride membrane (PVDF membrane; Millipore). The following primary antibodies were used: anti-GAPDH (Proteintech), anti-Tubulin (Proteintech), anti-LC3 (Novus Biologicals), anti-COXIV (Proteintech), anti-HSP60 (Proteintech), anti-TOM20 (Proteintech), anti-MFN2 (Proteintech), anti-TBK1 (Cell Signaling Technology), anti-OPTN (Proteintech) and anti-p-OPTN S177 (Cell Signaling Technology). The following secondary antibodies were used in our study: horseradish peroxidase-conjugated sheep anti-mouse and anti-rabbit antibodies (Jackson ImmunoResearch Laboratories). The proteins were visualized using an ECL detection kit (Thermo Fisher Scientific).

### 2.5 Immunofluorescent assay and live cell imaging

Immunofluorescent assay was performed similarly to the previous studies [19], [20], [21]. Briefly, the cells were fixed with 4% paraformaldehyde (PFA) at room temperature for 10 min, followed by permeabilization with 0.1% Triton X-100 for 10 min. The cells were first incubated with primary antibody in PBS at 4°C overnight and were washed with PBST for 5 min. Then the cells were incubated with secondary antibody for 2 h at room temperature. Finally, the immunostained or live cells were visualized using a Nikon inverted fluorescent microscope [22] or a Zeiss confocal microscope [23], [24], [25]. The following primary and secondary antibodies were used: anti-FLAG (Sigma), anti-HA (Santa Cruz Biotechnology), anti-HSP60 (Proteintech), anti-p-OPTN S177 (Cell Signaling Technology); Alexa Fluor 488-conjugated Affinipure Donkey Anti-mouse IgG (Proteintech), Alexa Fluor 647-conjugated Affinipure Donkey Anti-mouse IgG (Fcmacs Biotech) and Alexa Fluor 405-conjugated Affinipure Donkey Anti-rabbit IgG (Invitrogen). DAPI (Sigma) was used at room temperature for 5 min after the incubation of secondary antibodies. MitoTracker (Thermo Fisher Scientific), Propidium Iodide (PI; Beyotime) and Hoechst (Sigma) were used at 37°C for 20 min before the cells were fixed.

### 2.6 Statistical analysis

Photoshop (Adobe) software was used to perform immunoblot densitometric analyses of immunoblots from three independent experiments. The data were used for generating charts using Prism 7.0 (GraphPad Software) software. *P*-values and mean were indicated in figure legends. Two-way ANOVA was used to determine *P*-values in Fig. 2e and two-tailed *t*-test was used in other quantitative analyses. ImageJ (neuronJ) was used to measure neurite length and analyze the fluorescent intensity of mt-mKeima in Figs. 2b, d, 6c and d.

## 3 Results

### 3.1 Gefitinib enhances both autophagic and mitophagic fluxes in cells

**Fig. 1:**
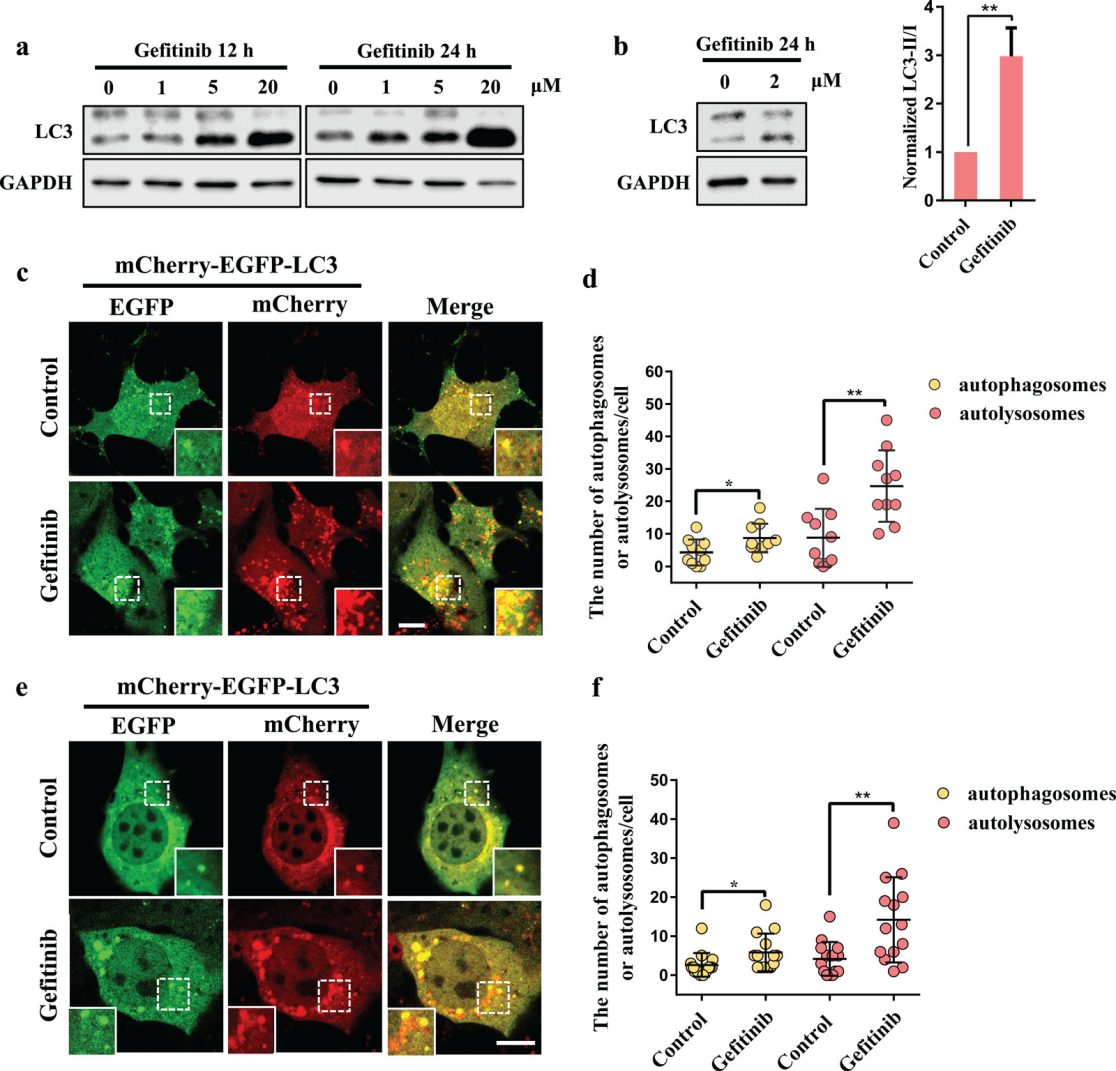
**Gefitinib increases general autophagy.** (a) HEK293 cells or (b) MEFs were treated with DMSO or the indicated concentration of gefitinib for 12 h or 24 h. Cell lysates were subjected to western blotting with antibodies against LC3 and GAPDH. Quantitative data show the normalized LC3 II/I as the mean ± SD, ^⁎⁎^*P* < 0.01, n = 3. (c) HEK293 cells or (e) MEFs were transfected with mCherry-EGFP-LC3 for 24 h and then treated with 2 μM gefitinib for 24 h. Fluorescence in living cells was visualized using confocal microscopy. Scale bars, 10 μm. The yellow dots indicate overlapping mCherry (red) and EGFP (green) signals that reflect autophagosomes. When autophagosomes fuse with acidic lysosomes, the EGFP signal quenches, showing red dots. (d and f) Quantification of autophagosomes (yellow dots) and autolysosomes (red dots) in (c) or (e). Data were collected from (d) 10 or (f) 14 cells as the mean ± SD, *P < 0.05, ^⁎⁎^*P* < 0.01.

Autophagy plays an important role in human diseases, including neurodegenerative diseases and cancer. According to a previous study, gefitinib can induce autophagy in cell lines in an EGFR-independent manner [26]. To clarify the change in autophagic flux after gefitinib treatment, we first examined the conversion of LC3-I to LC3-II, which is considered a marker of autophagosomes. Gefitinib treatment enhanced the numbers of autophagosomes in a dose-dependent manner in cells (Fig. 1a). Moreover, we found that gefitinib treatment at a clinically relevant concentration (2 μM) [27] also induced autophagosome formation in mouse embryonic fibroblasts (MEFs) with low EGFR expression levels (Fig. 1b). To further test the effect of gefitinib on autophagy, we used the mCherry-EGFP-LC3 fluorescent reporter to examine autophagic flux. In HEK293 and MEF cells, both autophagosome (yellow dots) and autolysosome (red dots) numbers were strikingly increased under gefitinib treatment, suggesting that gefitinib indeed enhanced autophagic flux (Fig. 1c–f). Taken together, our results suggest that autophagy was enhanced in cells after gefitinib treatment at clinically relevant concentrations.

**Fig. 2:**
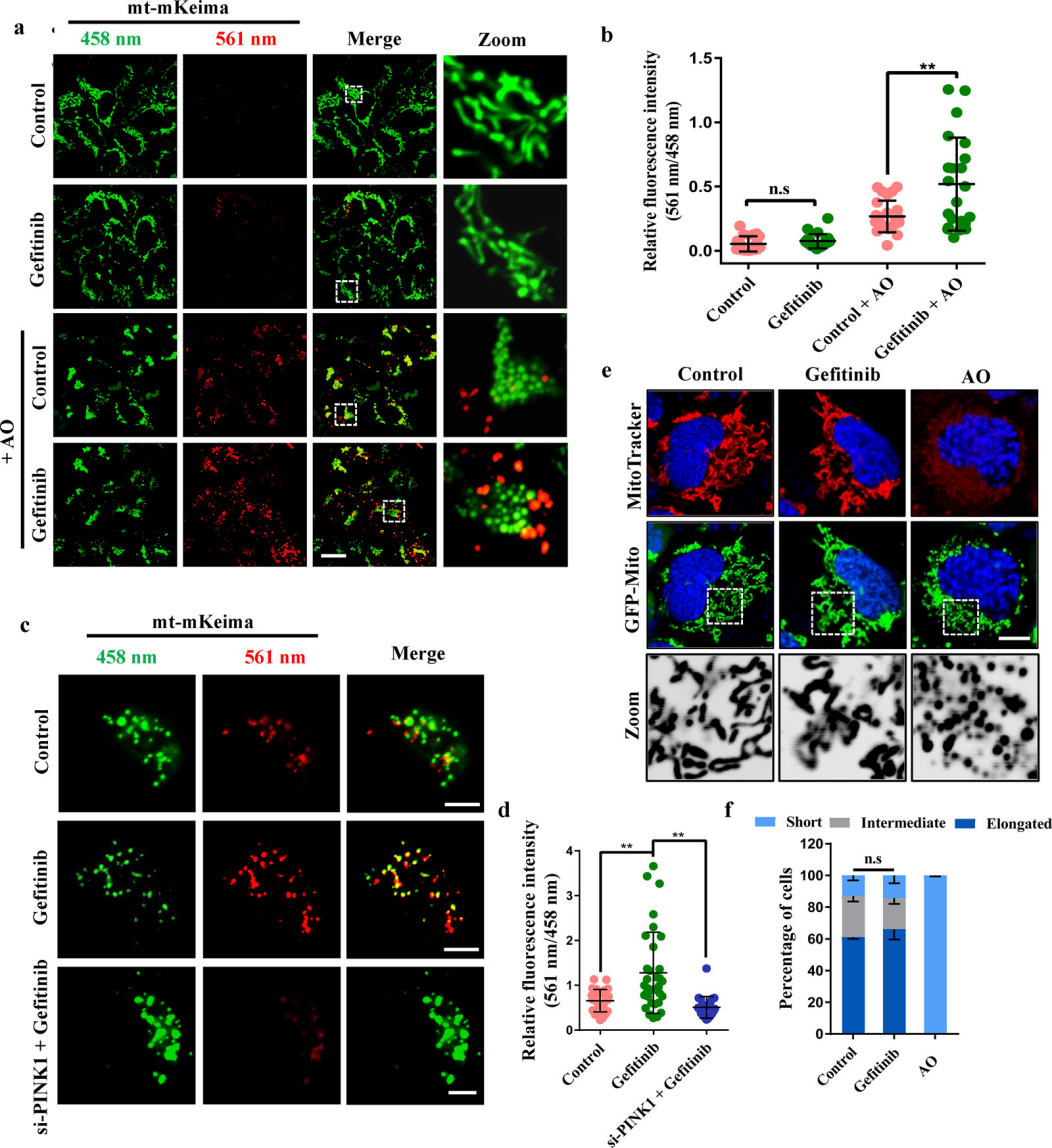
**Gefitinib enhances mitophagy without damaging mitochondria.** (a) HEK293 cells were transfected with GFP-Parkin and mt-mKeima. Four hours later, the cells were treated with DMSO or 2 μM gefitinib for 24 h and then incubated with 5 μg/ml AO for 4 h. Scale bar, 20 μm. (b) Quantification of the fluorescence intensity of the 561 (close to 586) nm/458 (close to 440) nm signal in (a). Data were collected from 18, 21, 25 or 22 cells in each sample and are shown as the mean ± SD, ^⁎⁎^*P* < 0.01, n.s., not significant. (c) A549 cells were transfected with PINK1 siRNA. Thirty-six hours after transfection, the cells were retransfected with GFP-Parkin and mt-mKeima. Four hours later, the cells were treated with DMSO or 2 μM gefitinib for 24 h and then incubated with 5 μg/ml AO for 14 h. Scale bars, 5 μm. (d) Quantification of the fluorescence intensity of the 561 nm/458 nm signal in (c). Data were collected from 26, 33 or 23 cells in each sample and are shown as the mean ± SD, ^⁎⁎^*P* < 0.01. (e) HEK293 cells were transfected with GFP-Mito and then treated with DMSO, 2 μM gefitinib for 24 h or 5 μg/ml AO for 4 h. The cells were stained with the potentiometric dye MitoTracker (red) and the nuclear dye Hoechst (blue) for 20 min before fixation. Scale bar, 10 μm. (f) The percentages of different mitochondrial lengths were quantified in (e). Quantitative data are represented as the mean ± SD, n.s., not significant, and over 100 cells were counted from three independent experiments.

Given that gefitinib could induce autophagy (Fig. 1), as previously reported [26,28,29], we wondered whether gefitinib could affect the selective form of autophagy, such as mitophagy. Therefore, we investigated the effect of gefitinib on PINK1/Parkin-mediated mitophagy in NSCLC and non-NSCLC cells. First, we used mt-mKeima, a specific mitophagy fluorescent reporter, to analyze mitophagic flux in gefitinib-treated cells [30]. mt-mKeima displays a 440 nm excitation range (green) under neutral pH, while it displays a 586 nm excitation range (red) when mitochondria are delivered to acidic lysosomes [13]. We treated Parkin-expressing HEK293 cells with inhibitors of mitochondrial complexes III and V (antimycin and oligomycin, hereafter referred to as AO) to induce mitophagy, and live-cell imaging experiments indicated that the mitophagic signal (red) was increased under gefitinib treatment in combination with AO (Fig. 2a and b). Meanwhile, gefitinib treatment alone did not induce mitophagy without additional mitochondrial damage, suggesting that gefitinib treatment did not harm mitochondria, and it may enhance mitophagy of damaged mitochondria but not healthy mitochondria (Fig. 2a and b). We further tested the effect of gefitinib on mitophagy in NSCLC cells, and our data showed that gefitinib significantly increased the mitophagy signal, which could be suppressed by PINK1 knockdown in A549 cells (Figs. 2c, d and S1a), indicating that gefitinib may widely enhance mitophagy driven by PINK1 in different types of cells.

We next used MitoTracker, a potentiometric dye, to analyze the mitochondrial membrane potential in gefitinib-treated cells. AO treatment, but not gefitinib treatment, strikingly reduced the mitochondrial membrane potential and led to fragmentation of mitochondria (Fig. 2e and f). Taken together, our data show that gefitinib treatment promotes PINK1/Parkin-mediated mitophagy in both NSCLC and non-NSCLC cells without damaging mitochondrial function and morphology.

### 3.2 Gefitinib promotes PINK1/Parkin-mediated mitophagy without affecting the mitochondrial recruitment of Parkin

**Fig. 3:**
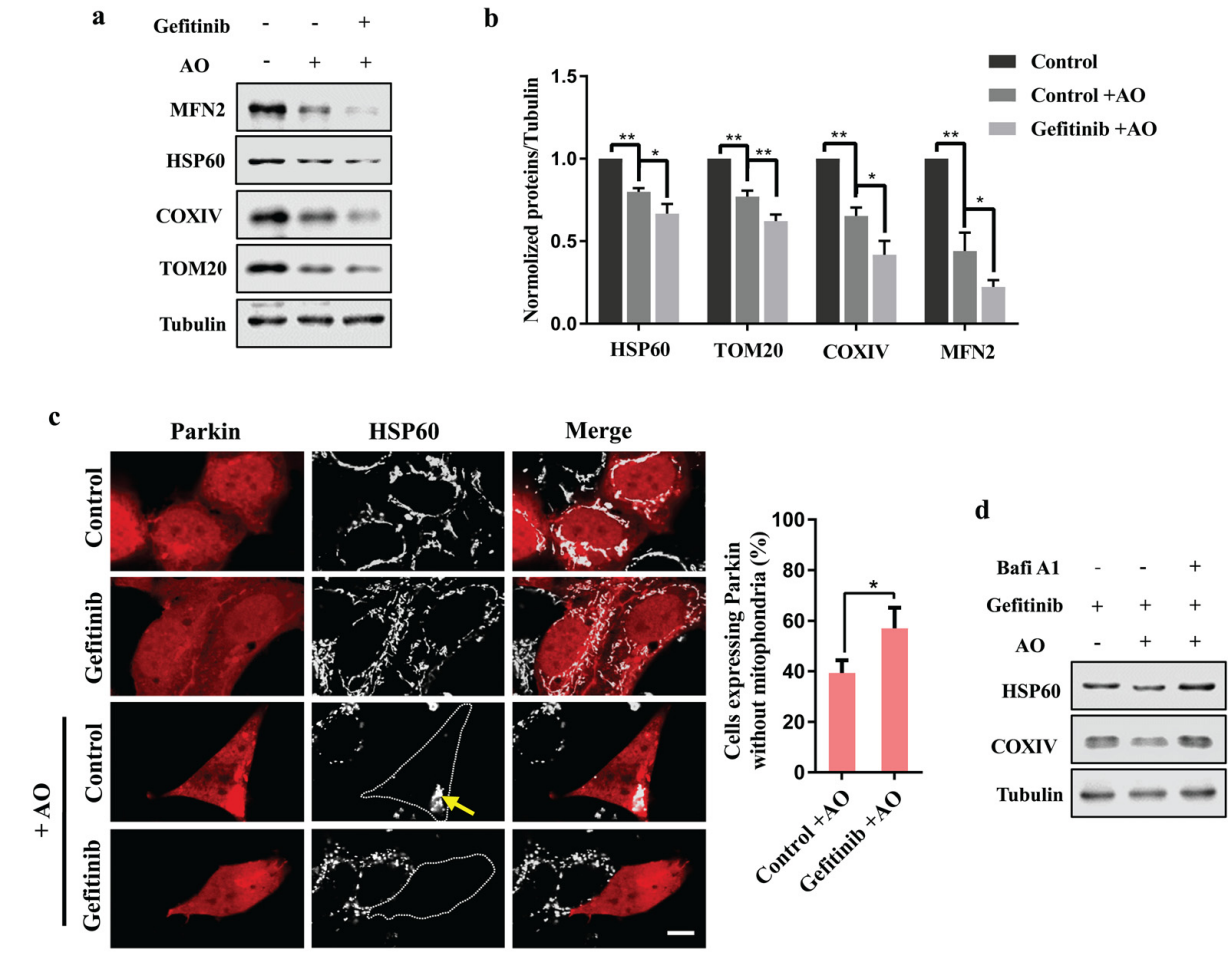
**Gefitinib promotes PINK1/Parkin-mediated autophagic clearance of mitochondria.** (a) HEK293 cells were transfected with mCherry-Parkin. Four hours later, the cells were treated with DMSO or 2 μM gefitinib for 24 h and then incubated with 1 μg/ml AO for 24 h. Cell lysates were analyzed by western blotting with antibodies against MFN2, HSP60, TOM20, COXIV and tubulin. (b) Quantification of MFN2, HSP60, TOM20 and COXIV protein levels in response to tubulin. Data from three independent experiments are represented as the mean ± SD, ^⁎⁎^*P* < 0.01, *P < 0.05, n = 3. (c) HEK293 cells were transfected with mCherry-Parkin. Four hours later, the cells were treated with DMSO or 2 μM gefitinib for 24 h and then incubated with 1 μg/ml AO for another 20 h. The cells were subjected to immunofluorescence with an anti-HSP60 antibody (white). Scale bar, 10 μm. The clearance of mitochondria was quantified in Parkin-positive cells. Quantitative data are represented as the mean ± SD, *P < 0.05, n = 3. (d) HEK293 cells were transfected with mCherry-Parkin. Four hours later, the cells were treated with 2 μM gefitinib for 24 h and then incubated with 1 μg/ml AO alone or in combination with 100 nM bafilomycin A1 (Bafi A1) for 24 h. Cell lysates were subjected to western blotting with the indicated antibodies.

To further explore the mechanism underlying gefitinib-induced mitophagy, we performed biochemical analysis to examine the clearance of various mitochondrial proteins, including the outer mitochondrial membrane proteins MFN2 and TOM20, the inner mitochondrial membrane protein COXIV and the mitochondrial matrix protein HSP60, upon gefitinib treatment (Fig. 3a and b). Gefitinib indeed promoted mitochondrial clearance in Parkin-positive but not Parkin-negative cells, which was confirmed by an immunofluorescence assay and suggested that gefitinib promoted PINK1/Parkin-mediated turnover of mitochondria (Fig. 3c). To further confirm gefitinib that promoted mitochondrial clearance via autophagy (mitophagy), we used bafilomycin A1 (Bafi A1), an inhibitor of autophagy, to examine the levels of mitochondrial proteins in gefitinib-treated cells. Mitochondrial clearance was inhibited upon Bafi A1 treatment (Fig. 3d). Taken together, our results suggest that gefitinib promotes PINK1/Parkin-mediated mitophagy.

**Fig. 4:**
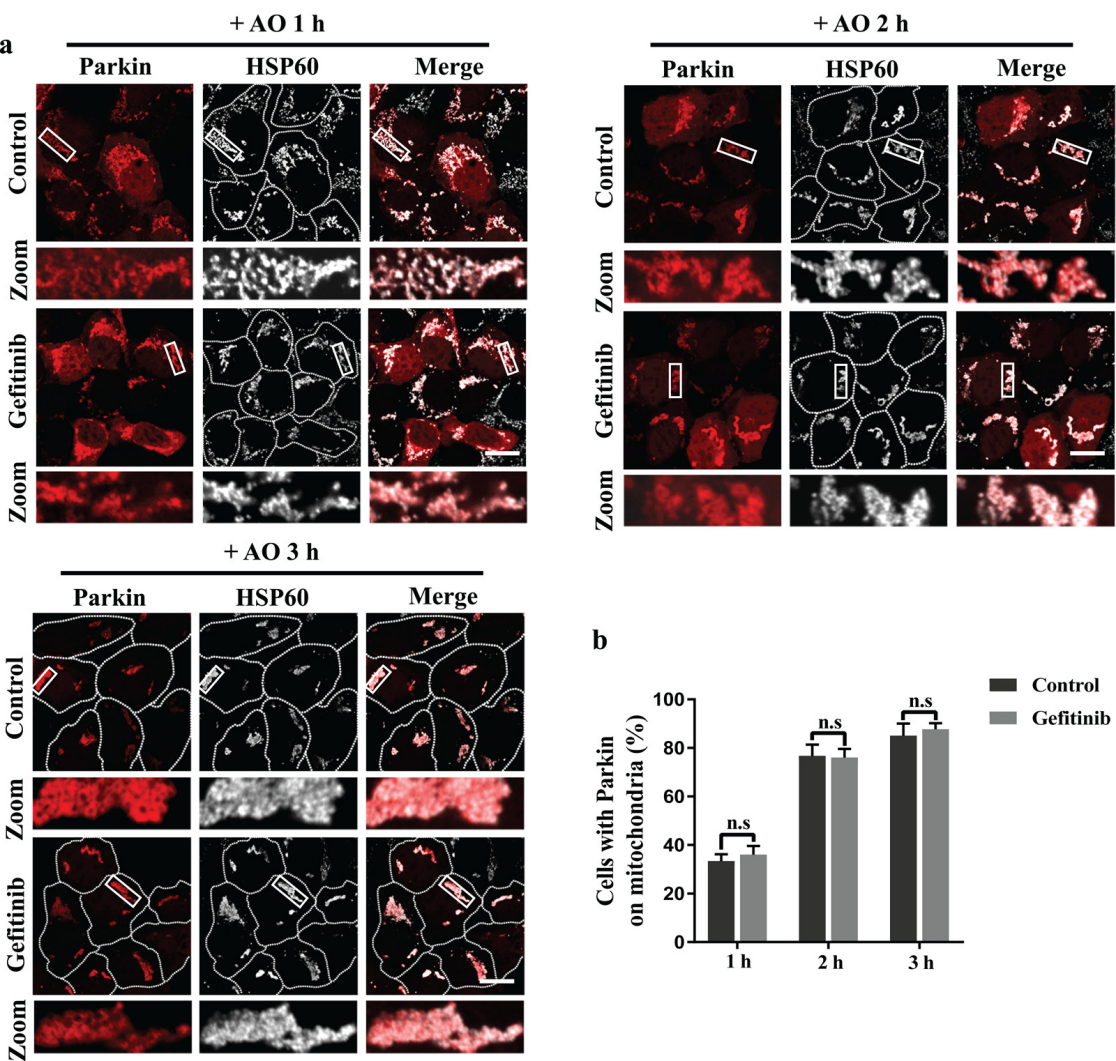
**Gefitinib has no effect on Parkin mitochondrial recruitment (upstream of mitophagy signaling).** (a) HEK293 cells were transfected with mCherry-Parkin. After 4 h, the cells were treated with DMSO or 2 μM gefitinib for 24 h and then incubated with 5 μg/ml AO for the indicated times. The cells were subjected to immunofluorescence with anti-HSP60 (white). Scale bars, 20 μm. (b) The percentages of Parkin on damaged mitochondria were quantified. Data are represented as the mean ± SD, n.s., not significant. Over 100 cells were counted from three independent experiments.

Since the E3 ubiquitin ligase Parkin is one of the key factors recruited to damaged mitochondria to trigger PINK1/Parkin-mediated mitophagy [31], we asked whether gefitinib promoted PINK1/Parkin-mediated mitophagy by increasing Parkin recruitment to damaged mitochondria. Our results showed that gefitinib had no influence on Parkin recruitment kinetics under AO treatment (Fig. 4a and b). Therefore, gefitinib may affect PINK1/Parkin-mediated mitophagy through other factors downstream of PINK1 and Parkin.

### 3.3 Gefitinib promotes mitochondrial transport of the mitophagy receptor OPTN and subsequent recruitment of autophagosomes to damaged mitochondria

**Fig. 5:**
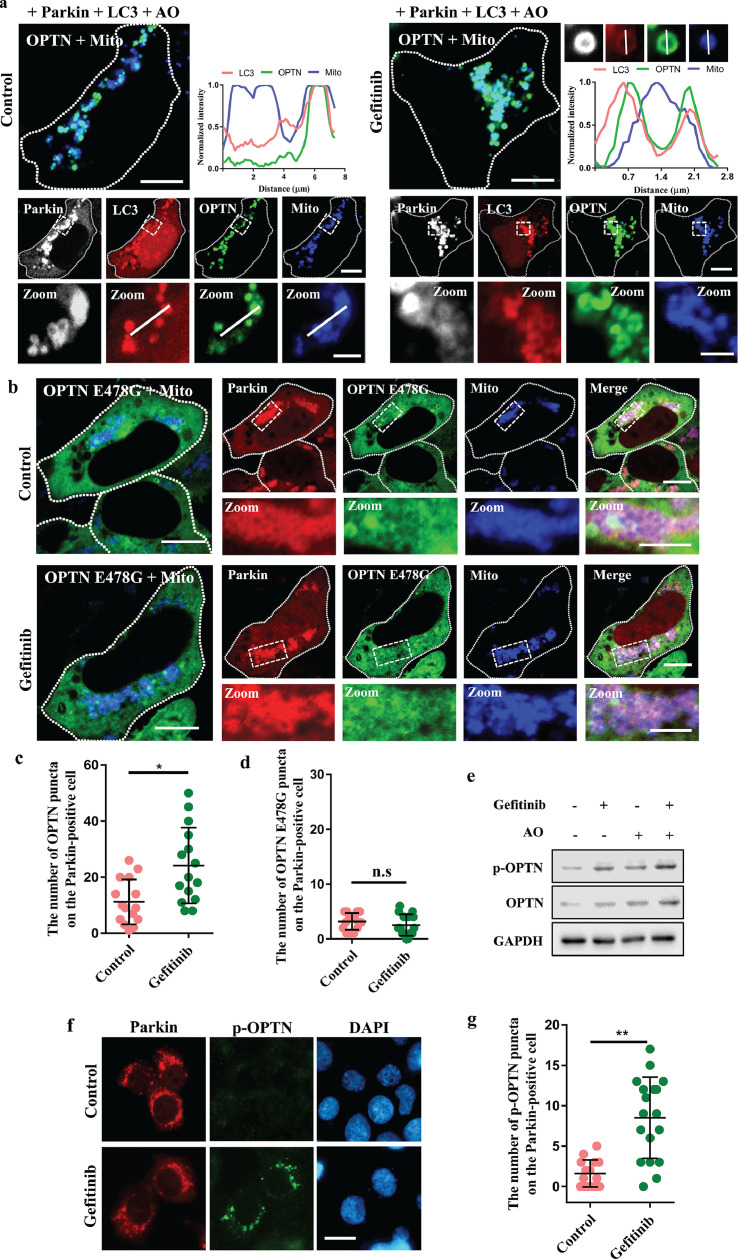
**Gefitinib enhances OPTN recruitment to damaged mitochondria and OPTN phosphorylation.** (a) HEK293 cells were transfected with FLAG-Parkin, mCherry-LC3, GFP-OPTN and BFP-Mito. The cells were treated with DMSO or 2 μM gefitinib for 24 h and then incubated with 5 μg/ml AO for 2 h. The cells were then subjected to immunofluorescence with an anti-FLAG antibody (white). The fluorescence intensity of representative images was quantified on the corresponding lines. Scale bars, 10 μm (upper panel) and 2 μm (lower panel). (b) HEK293 cells were transfected with mCherry-Parkin, GFP-OPTN E478G and BFP-Mito. After 4 h, the cells were treated with DMSO or 2 μM gefitinib for 24 h and then incubated with 1 μg/ml AO for 2 h. Scale bars, 10 μm (upper panel) and 5 μm (lower panel). (c) HEK293 cells were transfected with mCherry-Parkin, GFP-OPTN and BFP-Mito. After 4 h, the cells were treated with DMSO or 2 μM gefitinib for 24 h and then incubated with 5 μg/ml AO for 45 min. Quantification of the number of OPTN puncta on the Parkin-positive cells were represented as mean ± SD, *P < 0.05. Data were collected from 15 cells in each sample. (d) Quantification of the number of OPTN E478G puncta on the Parkin-positive cells are represented as mean ± SD, n.s., not significant. Data were collected from 15 cells in each sample. (e) HEK293 cells were transfected with mCherry-Parkin. After 4 h, the cells were treated with DMSO or 2 μM gefitinib for 24 h and then incubated with 5 μg/ml AO for 2 h. Cell lysates were analyzed by western blotting with antibodies against p-OPTN, OPTN and GAPDH. (f) HEK293 cells were transfected with mCherry-Parkin and HA-OPTN. After 4 h, the cells were treated with DMSO or 2 μM gefitinib for 24 h and then were incubated with 5 μg/ml AO for 1 h. The cells were subjected to immunofluorescence with anti-p-OPTN (green), and DAPI (blue) was used to indicate the nuclei. Scale bar, 20 μm. (g) Quantification of the number of p-OPTN puncta on the Parkin-positive cells are represented as mean ± SD, ^⁎⁎^*P* < 0.01. Data were collected from 15 or 18 cells in each sample.

Based on the fact that OPTN is recruited to damaged mitochondria after Parkin recruitment to facilitate the autophagosomal recognition of mitochondria as a mitophagy receptor [5,32], we asked whether OPTN could be affected by gefitinib during PINK1/Parkin-mediated mitophagy. To this end, we conducted an immunofluorescence assay to examine the recruitment of OPTN and autophagosomes (indicated by LC3). We found that gefitinib treatment increased the amounts of OPTN and LC3 colocalized with Parkin on damaged mitochondria (Fig. 5a, c). However, OPTN E478G, an ALS-linked mutant, was stalled in the cytoplasm and failed to translocate to damaged mitochondria under gefitinib treatment, although Parkin was strongly recruited to the mitochondria (Fig. 5b, d). Next, we examined the phosphorylation of OPTN, which was reported to regulate OPTN mitochondrial recruitment [9]. We found that gefitinib enhanced the level of p-OPTN S177 with or without AO treatment (Fig. 5e–g). Taken together, our results show that gefitinib enhances PINK1/Parkin-mediated mitophagy by increasing OPTN recruitment to damaged mitochondria.

### 3.4 Gefitinib promotes PINK1/Parkin-mediated mitophagy in an OPTN-dependent but not EGFR-dependent manner

**Fig. 6:**
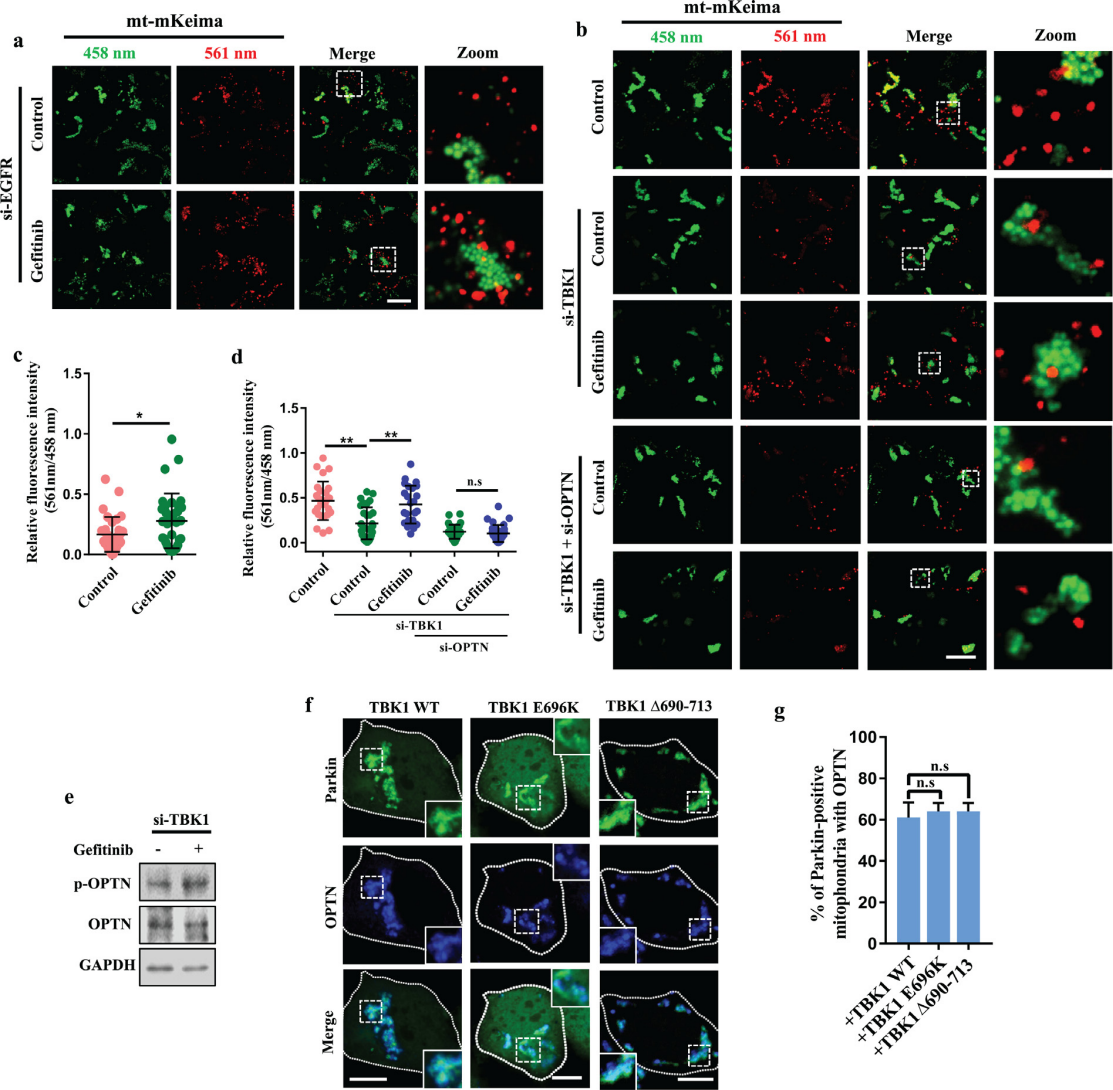
**Gefitinib enhances PINK1/Parkin-mediated mitophagy independent of EGFR and TBK1.** (a) HEK293 cells were first transfected with EGFR siRNA. Thirty-six hours after siRNA transfection, the cells were retransfected with GFP-Parkin and mt-mKeima. Four hours later, the cells were treated with DMSO or 2 μM gefitinib for 24 h and then 5 μg/ml AO for 4 h. Scale bar, 20 μm. (b) HEK293 cells were transfected with TBK1 alone or in combination with OPTN siRNA. After 48 h, the cells were transfected with GFP-Parkin and mt-mKeima. Four hours later, the cells were treated with DMSO or 2 μM gefitinib for 24 h and then incubated with 5 μg/ml AO for another 4 h. Scale bars, 20 μm. (c) Quantification of the fluorescence intensity of the 561 nm signal in (a). The quantitative data were collected from 32 cells in each sample, mean ± SD, **P < 0.01. (d) Quantification of the fluorescence intensity of 561 nm signal in (b). Data were collected from 26, 30, 24, 34 or 30 cells in each sample, mean ± SD, **P < 0.01, n.s., not significant. (e) HEK293 cells were treated with gefitinib and AO as in (a and b), then the cell lysates were collected and analyzed by western blotting with antibodies against p-OPTN, OPTN and GAPDH. (f) HEK293 cells were transfected with the TBK1 siRNA. Thirty-six hours later, the cells were retransfected with HA-OPTN and GFP-Parkin, along with FLAG-TBK1 WT, FLAG-TBK1 E696K or FLAG-TBK1 ∆690-713. After 4 h, the cells were treated with 2 μM gefitinib for 24 h, followed by 5 μg/ml AO treatment for 1.5 h. The cells were subjected to immunofluorescence with anti-HA (blue). Scale bars, 10 μm. (g) Quantification of the percentage of Parkin-positive mitochondria with OPTN in (f). Quantitative data are represented as mean ± SD, n.s., not significant, n = 3.

As gefitinib induced autophagy in EGFR-knockdown cells or EGFR low-expressing cells (Fig. 1) [26,27], we wondered whether gefitinib promoted PINK1/Parkin-mediated mitophagy in an EGFR independent manner. Thus, we investigated mitophagic flux using mt-mKeima in EGFR-knockdown cells. We found that gefitinib treatment increased the mitophagic signal in EGFR-deficient cells (Figs. 6a, c and S1b). TANK-binding kinase 1 (TBK1) is the upstream regulator of OPTN, and it is recruited along with OPTN to damaged mitochondria [7]. TBK1 phosphorylates OPTN at multiple sites to enhance OPTN mitochondrial recruitment and the recognition of damaged mitochondria by autophagosomes [7,9,33]. Next, we asked whether gefitinib induced mitophagy in a TBK1-dependent manner, and we found that gefitinib treatment promoted mitophagic flux in TBK1-deficient cells but not in OPTN-deficient cells (Figs. 6b, d, and S1c, d). Importantly, we found that gefitinib enhanced the level of p-OPTN S177 in TBK1-deficient cells. Moreover, OPTN recruitment was not affected by two ALS-causative TBK1 mutants, E696K and ∆690-713, indicating that gefitinib increases the phosphorylation and activation of OPTN independently of TBK1 kinase (Fig. 6e–g). Taken together, these data suggest that gefitinib enhances mitophagy by regulating mitochondrial recruitment and activation of OPTN, the key mitophagy receptor that can regulate mitophagy downstream of TBK1, in an EGFR-independent manner.

### 3.5 Gefitinib protects TBK1-deficient neurons by restoring impeded mitophagy

**Fig. 7:**
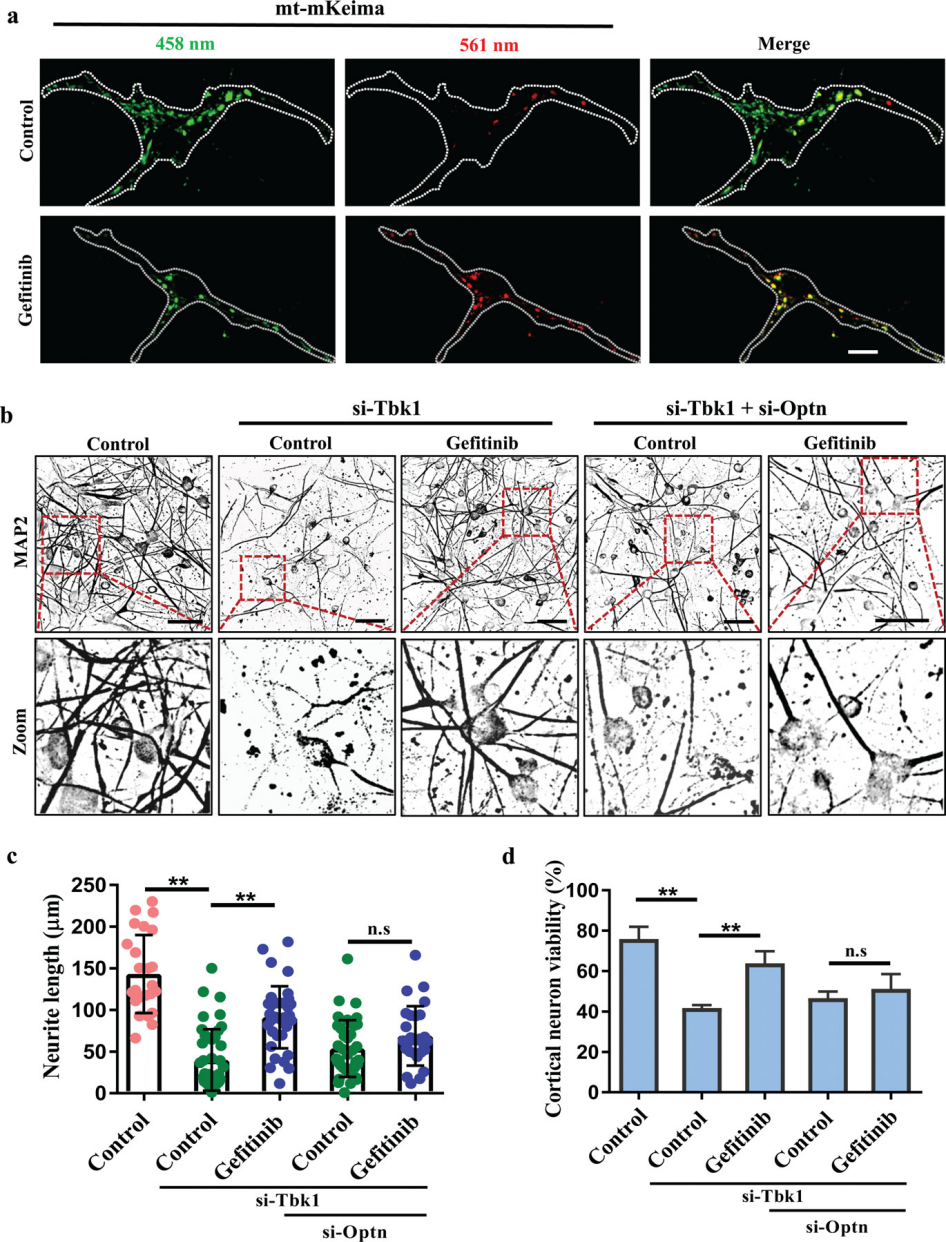
**Gefitinib protects primary cortical neurons under Tbk1 loss by inducing mitophagy.** (a) Primary cortical neurons were infected with mt-mKeima. After 48 h, the neurons were treated with DMSO or 50 nM gefitinib for 24 h and then incubated with 1 μg/ml AO for 4 h. Scale bar, 10 μm. (b) Primary cortical neurons were transfected with Tbk1 (mouse) siRNA alone or in combination with Optn (mouse) siRNA. Forty-eight hours after transfection, the cells were treated with DMSO or 50 nM gefitinib for 24 h and then with 1 μg/ml AO for 4 h. The live cells were stained with Hoechst and PI and then subjected to immunofluorescence with an anti-MAP2 antibody. Scale bars, 50 μm. (c) The neurite length of each group was measured by ImageJ (NeuronJ) in (b). Quantitative data were collected from 25, 42, 37, 36 or 27 cells in each sample, mean ± SD, ^⁎⁎^*P* < 0.01, n.s., not significant. (d) The viability of cortical neurons was quantified by PI/Hoechst. Quantitative data were counted from over 300 cells for each sample, mean ± SD, ^⁎⁎^*P* < 0.01, n.s., not significant.

Given that TBK1 deficiency leads to neurodegenerative disorders, including ALS [34,35], and impaired mitophagy [7,9,33], we wondered whether gefitinib may have a protective effect against TBK1 deficiency-induced neurotoxicity. We examined mitophagic flux using mt-mKeima in primary cultured cortical neurons and found that gefitinib treatment significantly increased mitophagic flux in Tbk1 (mouse homolog of human TBK1)-deficient neurons (Fig. 7a). In addition, we used MAP2, a neuronal marker, to detect the neuronal morphology and neurite outgrowth that were impaired in TBK1-deficient neurons (Fig. 7b–d). Gefitinib treatment significantly restored neurite outgrowth and neuronal viability in Tbk1-deficient but not Tbk1 and Optn (mouse homolog of human OPTN) double-deficient neurons (Fig. 7b–d). Overall, our data suggest that gefitinib treatment is beneficial for neuronal survival when mitophagy is impaired due to the loss of Tbk1.

## 4 Discussion

Although increasing evidence has shown that mitophagy is protective for neuronal cells in neurodegenerative diseases, including PD and ALS, it is currently still unclear whether induction of mitophagy is beneficial for TBK1-mediated ALS. Our current findings suggest that OPTN activity modulates neurite morphology in a neuronal culture model of ALS with TBK1 deficiency and decreased mitophagy. Gefitinib treatment reduced neurotoxicity in this model (Fig. 7).

Pharmacological modulation of mitophagy is considered potentially beneficial for the treatment of human diseases such as neurodegeneration and cancer [12]. For example, induction of mitophagy by the small molecule compounds kinetin, DFP, Urolithin A, UMI-77 and an inhibitor of USP30 at multiple steps in the mitophagy pathway has been considered a potential treatment for neurodegenerative diseases, including PD and Alzheimer's disease (AD) [36], [37], [38], [39], [40], [41]. However, it is rare to see FDA-approved drugs as potential inducers of mitophagy. In addition, given that ALS-linked TBK1 deficiency may induce neurodegeneration by blocking downstream mitophagy signaling, our study not only highlights the FDA-approved drug gefitinib as a potential clinical treatment for TBK1-mediated ALS but also provides new insights into the downstream mitophagic regulator OPTN as a new target for pharmacology research in the future.

Interestingly, gefitinib induces mitophagy in an EGFR-independent manner (Fig. 6), indicating that gefitinib has multiple biological targets in cells. We found that OPTN mitochondrial recruitment and activity were significantly enhanced after gefitinib treatment (Fig. 5), and we speculate that there are three possibilities: (1) gefitinib directly binds to OPTN and facilitates the translocation of OPTN to damaged mitochondria; (2) gefitinib directly interacts with the OPTN-binding partner to enhance OPTN mitochondrial recruitment; and (3) gefitinib targets unknown regulators to indirectly affect the activity of OPTN. Taken together, our study highlights OPTN as a novel and important pharmacological target for manipulating mitophagy and suggests that activation of OPTN might be a new strategy to treat neurodegenerative diseases.

## 5 Conclusion

**Fig. 8:**
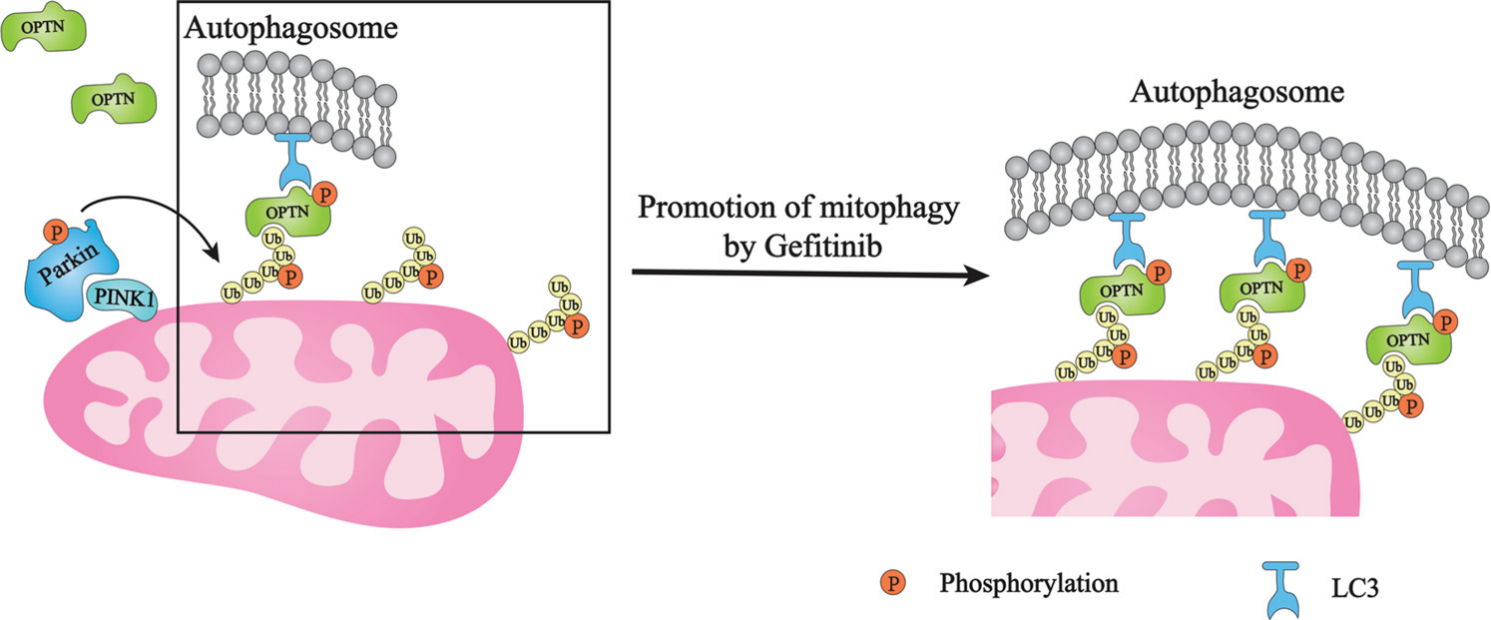
**Schematic model of the present study.** During PINK1/Parkin-mediated mitophagy, PINK1 and Parkin initiate the ubiquitination of damaged mitochondria, and ubiquitinated mitochondria are recognized by the autophagy receptor OPTN. The present study suggests that gefitinib treatment promotes OPTN phosphorylation and mitochondrial recruitment, thereby facilitating the autophagosomal recognition of damaged mitochondria to ensure efficient mitophagy.

The present study includes the following findings, which are summarized in the schematic model (Fig. 8): (1) gefitinib can induce both general autophagy and selective autophagy of mitochondria (mitophagy) independent of EGFR (Fig. 1, Fig. 2, Fig. 3 and 6); (2) gefitinib enhances mitophagy without damaging mitochondria (Fig. 2); (3) gefitinib targets downstream OPTN signaling but not upstream PINK1/Parkin signaling in the mitophagy pathway (Fig. 4, Fig. 5, Fig. 6); and (4) the upregulation of PINK1/Parkin-mediated mitophagy by gefitinib has a beneficial effect on Tbk1-deficient neurons (Fig. 7). Taken together, our study suggests that OPTN plays an important role in gefitinib-induced mitophagy and can be considered a potential therapeutic target in ALS.

## Declaration of Competing Interest

The authors declare that they have no conflicts of interest in this work.

## Appendix Supplementary materials

Image, application 1
