# Supplementary material for: Gefitinib facilitates PINK1/Parkin-mediated mitophagy by enhancing mitochondrial recruitment of OPTN
Source: Fundam Res. 2022 Mar 3;2(5):807–16. doi: 10.1016/j.fmre.2021.12.017 (PMC11197598; doi:10.1016/j.fmre.2021.12.017)

Supplementary Material

Gefitinib facilitates PINK1/Parkin-mediated mitophagy through enhancing mitochondrial recruitment of OPTN

Ningning Li[1], Shan Sun[1], Guoqiang Ma[1], Hongyu Hou[1], Qilian Ma[1], Li Zhang[3], Zengli Zhang[2, *], Hongfeng Wang[1, *], Zheng Ying[1, *]

[1]Jiangsu Key Laboratory of Neuropsychiatric Diseases and College of Pharmaceutical Sciences, Soochow University, Suzhou, Jiangsu 215123, China
[2]Department of Respiratory and Critical Care Medicine, The Second Affiliated Hospital of Soochow University, Suzhou, Jiangsu 215004, China
[3]Key Laboratory of Nuclear Medicine, Ministry of Health, Jiangsu Key Laboratory of Molecular Nuclear Medicine, Jiangsu Institute of Nuclear Medicine, Wuxi, Jiangsu 214063, China

*Address correspondence to:
Zheng Ying, Jiangsu Key Laboratory of Neuropsychiatric Diseases and College of Pharmaceutical Sciences, Soochow University, Suzhou, Jiangsu 215123, China. E-mail addresses: zheng.ying@suda.edu.cn
Hongfeng Wang, Jiangsu Key Laboratory of Neuropsychiatric Diseases and College of Pharmaceutical Sciences, Soochow University, Suzhou, Jiangsu 215123, China. E-mail addresses: wanghongfeng@suda.edu.cn
Zengli Zhang, Department of Respiratory and Critical Care Medicine, The Second Affiliated Hospital of Soochow University, Suzhou, Jiangsu 215004, China. E-mail addresses: zenglizhang@126.com

Supplementary Material

Fig. S1 The knockdown efficiency of the RNAi experiments. (a) HEK293 cells were transfected with the PINK1 siRNA for 72 h. Cell lysates were subjected to western blotting with antibodies against PINK1 and GAPDH. (b) HEK293 cells were transfected with the EGFR siRNA for 72 h. The EGFR mRNA level was examined by qPCR. Quantitative data were represented as mean ± SD, $*P<0.05$, n=3. (c) and (d) HEK293 cells were transfected with the TBK1 siRNA or OPTN siRNA for 72 h. Cell lysates were subjected to western blotting with antibodies against TBK1 or OPTN and GAPDH.

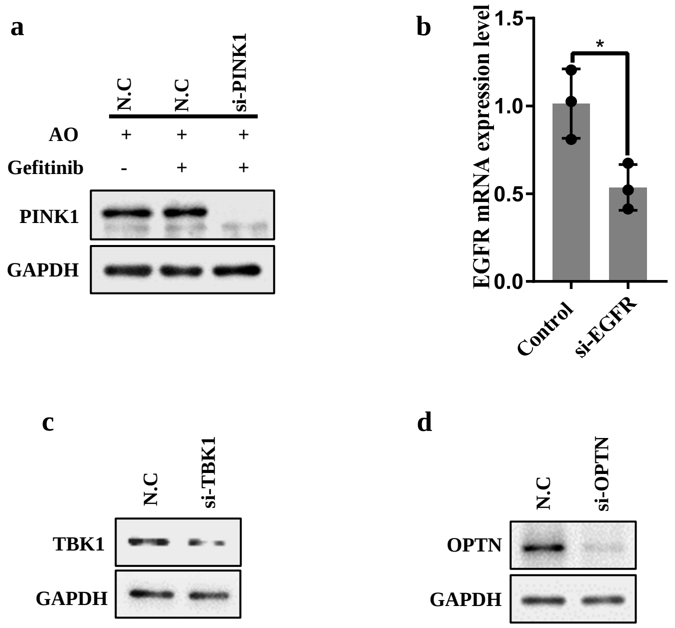

Supplement: Supplementary file 1 [file mmc1.pdf]
